# GroupICA dual regression analysis of resting state networks in a behavioral variant of frontotemporal dementia

**DOI:** 10.3389/fnhum.2013.00461

**Published:** 2013-08-26

**Authors:** Riikka Rytty, Juha Nikkinen, Liisa Paavola, Ahmed Abou Elseoud, Virpi Moilanen, Annina Visuri, Osmo Tervonen, Alan E. Renton, Bryan J. Traynor, Vesa Kiviniemi, Anne M. Remes

**Affiliations:** ^1^Department of Neurology, Institute of Clinical Medicine, University of OuluOulu, Finland; ^2^Department of Neurology, Oulu University HospitalOulu, Finland; ^3^Department of Radiology, Institute of Clinical Medicine, University of OuluOulu, Finland; ^4^Neuromuscular Diseases Research Unit, Laboratory of Neurogenetics, National Institute on Aging, National Institutes of HealthBethesda, MD, USA; ^5^Department of Neurology, Institute of Clinical Medicine, University of Eastern FinlandKuopio, Finland; ^6^Department of Neurology, Kuopio University HospitalKuopio, Finland

**Keywords:** default, resting state, functional MRI, dorsal attention network, frontotemporal dementia

## Abstract

Functional MRI studies have revealed changes in default-mode and salience networks in neurodegenerative dementias, especially in Alzheimer's disease (AD). The purpose of this study was to analyze the whole brain cortex resting state networks (RSNs) in patients with behavioral variant frontotemporal dementia (bvFTD) by using resting state functional MRI (rfMRI). The group specific RSNs were identified by high model order independent component analysis (ICA) and a dual regression technique was used to detect between-group differences in the RSNs with *p* < 0.05 threshold corrected for multiple comparisons. A *y*-concatenation method was used to correct for multiple comparisons for multiple independent components, gray matter differences as well as the voxel level. We found increased connectivity in several networks within patients with bvFTD compared to the control group. The most prominent enhancement was seen in the right frontotemporal area and insula. A significant increase in functional connectivity was also detected in the left dorsal attention network (DAN), in anterior paracingulate—a default mode sub-network as well as in the anterior parts of the frontal pole. Notably the increased patterns of connectivity were seen in areas around atrophic regions. The present results demonstrate abnormal increased connectivity in several important brain networks including the DAN and default-mode network (DMN) in patients with bvFTD. These changes may be associated with decline in executive functions and attention as well as apathy, which are the major cognitive and neuropsychiatric defects in patients with frontotemporal dementia.

## Introduction

Frontotemporal lobar degeneration (FTLD) is a clinically and pathologically heterogeneous syndrome characterized by a progressive decline in behavior and/or language associated with degeneration of the frontal and anterior temporal lobes (Neary et al., 2005). FTLD is the second most frequent neurodegenerative disease leading to early onset dementia after Alzheimer's disease (AD). The most common clinical presentation of FTLD is behavioral-variant frontotemporal dementia (bvFTD). The prominent symptoms in bvFTD are a gradual decline in social behavior with loss of insight and decline in executive functions instead of typical memory problems.

Resting state functional MRI (rfMRI) can be used to study the intrinsic connectivity of brain networks in task-free settings by mapping temporally synchronous, spatially distributed, spontaneous low frequency (<0.08 Hz) blood oxygen level-dependent (BOLD) signal fluctuations (Fox and Raichle, 2007). rfMRI reports have revealed a number of functional networks in the brain, but mainly two major intrinsic connectivity networks, the default-mode network (DMN) and salience, have been studied in neurodegenerative diseases. The DMN is a posterior network that consists of the hippocampi, posterior cingulate cortex/precuneus, lateral parietal regions, and the rostromedial prefrontal cortex (Raichle et al., 2001). Regions of the DMN seem to participate in episodic memory and visuospatial imagery (Zhou et al., 2010). The salience network is a large anterior network that consists of the anterior cingulate cortex (ACC), orbital frontoinsula (FI), amygdala, and striatum and is related to socially-emotionally relevant information processing (Seeley et al., 2007a,b). Decreased connectivity in the DMN is consistently associated with AD (Greicius et al., 2004; Zhang et al., 2009; Gili et al., 2011), while reduction of connectivity in the salience network has been usually found in patients with bvFTD. However, the findings in patients with bvFTD are controversial (Zhou et al., 2010; Whitwell et al., 2011; Borroni et al., 2012). Disruption in other functional networks such as the dorsal attention network (DAN) has been found in AD (Li et al., 2012).

Previous rfMRI analyses have been done using relatively low model order (<30) independent component analysis (ICA) estimated from a single subject or group analyses (Zhou et al., 2010; Whitwell et al., 2011; Borroni et al., 2012). Detecting multiple networks from 1.5 T BOLD data from a single subject is not optimal in all occasions. Instead, group ICA offers much more robust delineation of identifiable networks (Kiviniemi et al., 2009; Smith et al., 2009; Abou-Elseoud et al., 2010). Also, the use of a higher model order in ICA detects more networks under detected at low model orders and introduces sub-network delineation of resting state networks (RSNs) at more detailed level separating noise more appropriately (Abou Elseoud et al., 2011, 2012). The problem with the more robust group ICA networks is still the challenge of acquiring accurate representations of networks from the individual level for a statistical group analysis.

The dual regression method has been introduced in order to overcome statistical inference problems (Filippini et al., 2009). Zuo and co-workers found that not only is the temporal concatenated ICA dual regression approach reliable, it produces more robust results than template matching ICA performed on an individual level (Zuo et al., 2010). This variability at the individual level seems to arise from the non-stationarity of the networks themselves, rather than the ICA method instability (Zuo et al., 2010; Kiviniemi et al., 2011; Hutchison et al., 2013). Moreover, group-level averaging seems to increase matching accuracy to RSN templates as well.

In order to obtain robust whole brain cortex network status with all relevant functional RSNs one still has to account for the multiple comparison problem introduced by the usage of several ICs. We have recently developed a *y*-concatenation method for correcting for multiple comparisons for multiple IC components as well as at the voxel level (Abou Elseoud et al., 2012). This correction enables the detection of statistically significant alterations between groups in all analysed RSNs of the brain cortex.

In bvFTD executive dysfunction is the prominent neuropsychological finding, and neurodegeneration is seen in frontal, temporal and insular areas including parts of the salience network (Neary et al., 1998, 2005; Rosen et al., 2002; Seeley et al., 2007a,b; Whitwell et al., 2009). Thus, we hypothesized to find decreased connectivity in the salience network associated with changes in executive networks. Our aim was to obtain a robust, fully data driven measure of the functional connectivity changes of all RSNs covering the whole brain. For this we performed time concatenated temporal ICA with a dual regression approach with y-concatenation statistics of bvFTD data compared to controls.

## Materials and methods

### Patients

The patient group included 19 cases with bvFTD (nine male). A clinical diagnosis of bvFTD was made according to the criteria of Lund and Manchester (Neary et al., 1998; Rascovsky et al., 2011). Patients presenting other types of FTLD phenotype such as progressive aphasia and semantic dementia were excluded. All patients were examined in Oulu University Hospital at the Memory outpatient clinic of the Department of Neurology. The mean age at examination was 60.3 years (range 47–77 years). MMSE (Mini-Mental State Examination) score was on average 24.1 (17–30). Neuropsychological examination was performed within 6 months of the fMRI scan of each patient. Impairments in memory, language, executive, and visuospatial functions were then rated in three stages (absent, mild to moderate, severe). The declines in attention and executive functions were the most prominent findings in all of the patients, but there was also significant deterioration in other cognitive domains. Patients' reading and writing skills were normal. Dyspraxia was not present, and motor speed and control were at a normal level. Apathy was the most prevalent neuropsychiatric symptom (74%), but depression and irritability was also common (50%). The severity of the disease was rated as mild in nine patients, moderate in nine patients and severe in one patient (Piguet et al., 2011). Medications for neuropsychiatric symptoms were used in some of the patients (acetylcholinesterase inhibitors in three patients, memantine in two, neuroleptics in eight and valproate in three). Positive family history for any type of dementia was seen in 52.6% (*n* = 10) of patients. DNA samples were available from ten patients and five of them carried a C9ORF72 repeat expansion (Renton et al., 2011). Mutations in progranulin or microtubule-associated protein tau genes were not found in any of the patients.

Age and gender-matched controls (*n* = 19) were also examined. Mean age at examination was 57.8 years (range 50–70). No psychiatric or neurological disorders or medications affecting the central nervous system were allowed. Beck's depression inventory (BDI) score was mean 2.7 (range 0–10) and MMSE mean was 28.9 (range 26–30).

Written informed consent was obtained from all of the patients or their guardians. The research protocol was approved by the Ethics Committee of the Northern Ostrobothnia Hospital District.

### Imaging protocol

Resting-state BOLD data were collected on a GE Signa 1.5 T MRI scanner with an 8-channel parallel imaging-coil ASSET system (acceleration factor × 2) with an EPI GRE sequence (TR 1800 ms, TE 40 ms, 285 time points, 28 oblique axial slices, slice thickness 4 mm, inter-slice space 0.4 mm, covering the whole brain with an FOV of 25.6 cm × 25.6 cm with 64 × 64 matrix, and a flip angle of 90°). Hearing was protected using ear plugs and motion was minimized by using soft pads fitted over the ears. The subjects were instructed to simply lay still inside the scanner with their eyes closed, think of nothing in particular and not to fall asleep. High-resolution T1-weighted 3D FSPGR BRAVO images were taken in order to obtain anatomical images for co-registration of the fMRI data to the standard space coordinates and to investigate voxel-wise changes in the gray matter volume.

### Structural analysis

Structural data were analysed with FSL-VBM (www.fmrib.ox.ac.uk/fsl), a voxel-based morphometry style analysis (Ashburner and Friston, 2000; Good et al., 2001). Firstly, structural images were brain-extracted using BET (Smith, 2002). Next, tissue-type segmentation was carried out using FAST4 (Zhang et al., 2001). The resulting gray matter partial volume images were aligned to MNI152 standard space using nonlinear registration FNIRT in FSL (www.fmrib.ox.ac.uk/analysis/techrep), which uses a b-spline representation of the registration warp field (Rueckert et al., 1999). The resulting images were averaged to create a study-specific template, to which the native gray matter images were then non-linearly re-registered. The registered partial volume images were then modulated to correct for local expansion or contraction by dividing by the Jacobian of the warp field. The modulated segmented images were then smoothed with an isotropic Gaussian kernel with a sigma of 3 mm. Finally, voxelwise GLM was applied using FSL's randomize, which is a permutation-based non-parametric testing, correcting for multiple comparisons across space with *p* < 0.05 threshold.

### fMRI data pre-processing

Data pre-processing was carried out with FSL tools. Head motion in the fMRI data was corrected using multi-resolution rigid body co-registration of volumes, as implemented in the MCFLIRT software (Jenkinson et al., 2002). Brain extraction was carried out for motion corrected BOLD volumes with optimization of the deforming smooth surface model, as implemented in the BET software (Smith, 2002). This procedure was verified with visual inspection of the extraction result. The resulting image data was used as a mask for a secondary brain extraction. Multi-resolution affine co-registration as implemented in the FLIRT software was used to co-register fMRI volumes to 3D FSPGR volumes of the corresponding subjects and further the 3D FSPGR volumes to the MNI152 standard space. The images were transformed to 4 mm cubic voxels with 5 mm FWHM smoothing. There were no differences in head motion parameters in absolute [FTD (0.30 ± 0.12 mm) vs. control (0.26 ± 0.13 mm, *t*-test *p* = 0.36)] or relative [FTD (0.07 ± 0.03 mm) vs. controls (0.06 ± 0.03 mm, *t*-test *p* = 0.34)] between the study groups. Maximum absolute (2.9 mm) and relative (3.1 mm) head motion were below the voxel size in all subjects.

### Functional connectivity analysis

**Figure 1:**
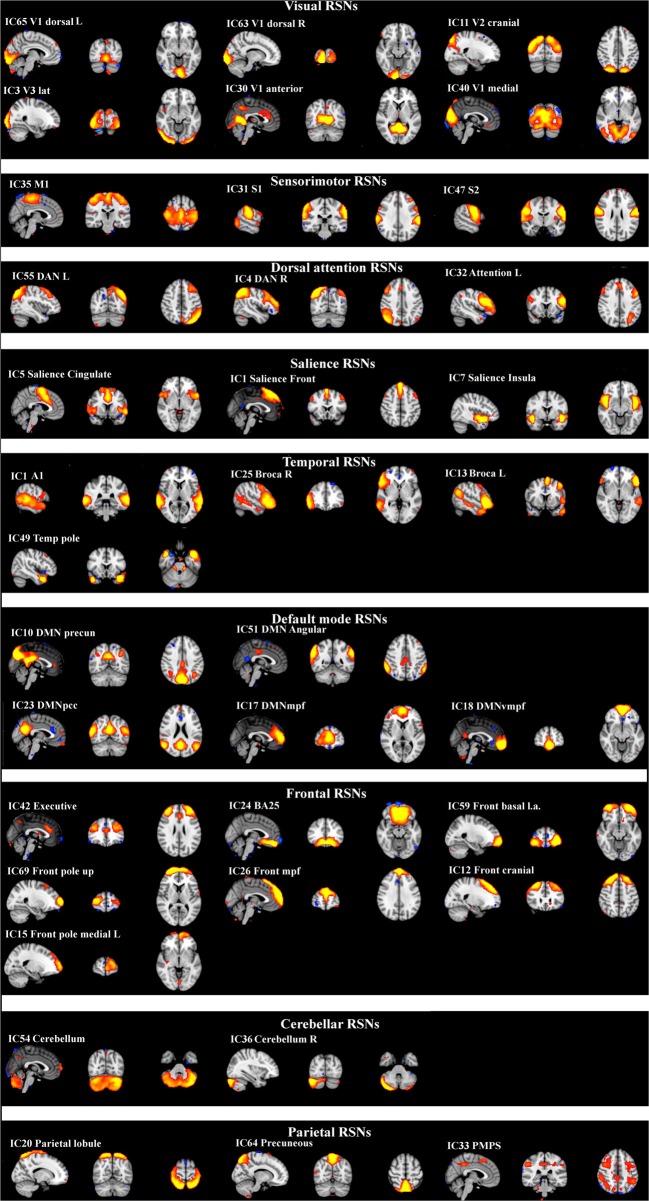
**Resting-state networks from the control group identified as anatomically and functionally classical RNSs and which were used for the dual regression analysis.** The normal resting-state networks are shown in FSL red-yellow color encoding using a 3 < *z*-score < 9 threshold.

ICA analysis has been conducted as previously described (Abou Elseoud et al., 2011). Briefly, ICA analysis was carried out using FSL 4.1.4 MELODIC software implementing probabilistic independent component analysis (PICA) (Beckmann and Smith, 2004). A multisession temporal concatenation tool in MELODIC was used to perform PICA related pre-processing and data conditioning in the group analysis setting. Spatial ICA using 70 independent component maps (IC maps) was applied to detect RSNs from the control group. Control group data was chosen for two reasons: Firstly, our experience is that a combined groupICA having both cases and controls produces averaged maps of both groups which are then less sensitive in detecting differences between the groups in dual regression. Secondly, control data groupICA results are more robust match with previous healthy control data groupICA templates without disease-related alterations (Kiviniemi et al., 2009; Smith et al., 2009). Variance normalization was used. The IC maps were thresholded using an alternative hypothesis test based on fitting a Gaussian/gamma mixture model to the distribution of voxel intensities within spatial maps and controlling the local false-discovery rate at *P* < 0.5 (Beckmann and Smith, 2004; Beckmann et al., 2005). Thirty-six RSNs were identified as anatomically and functionally classical RSNs upon visual inspection by an experienced neuroradiologist (VK) using previously described criteria (Kiviniemi et al., 2009; Smith et al., 2009; Abou-Elseoud et al., 2010). The 36 RSNs are presented in Figure 1.

The analysis for the differences between groups was carried out using an FSL dual regression technique that allows for voxel-wise comparisons of resting-state fMRI (Filippini et al., 2009; Littow et al., 2010; Veer et al., 2010; Abou Elseoud et al., 2011). This involves (A) using the group-ICA spatial maps in a linear model fit against the separate fMRI data sets, resulting in matrices (time-course matrices) describing the temporal dynamics for each component and subject, and (B) using these time-course matrices to estimate subject-specific spatial maps. The ICA template for the dual regression was selected from the healthy control data. The dual regression analysis was performed both with and without variance normalization (FSL414 dual_regression command with des norm-option 1 or 0, respectively), since the results have a different emphasis on the spatial or amplitude of the BOLD signal depending on normalization (Allen et al., 2012a,b). With variance normalization, the dual regression reflects differences in both activity and spatial spread of the RSN. Without normalization, only spatial alterations are reflected in principle.

As a statistical analysis the different component maps are collected across subjects into single 4D files (1 per original ICA map) and tested voxel-wise for statistically significant differences between the groups using FSL randomize nonparametric permutation testing, with 5000 permutations, using a threshold-free cluster enhanced (TFCE) technique to control for multiple comparisons (Nichols and Holmes, 2002). The hypothesized differences between groups were calculated using a *p* < 0.05 threshold with voxel-wise changes in the FSL randomize tool. A newly developed inter-IC concatenation technique was used in order to control for multiple comparisons across the detected IC differences (Abou Elseoud et al., 2012). After discarding noise, motion and other artifact components, all the 36 RSN ICs were concatenated in the y-direction and fed to the FSL randomize tool as a joint dataset. After 5000 permutations the resulting datasets were separated with fslroi and thresholded for *p* < 0.05 correcting for type I error for the selected multiple ICs. As a new step, a gray matter regressor image was also fed in a similar fashion such that it was concatenated in the y-direction to control for gray matter differences with FSL randomize using—vxl and—vxf options.

The Juelich histological atlas incorporated in FSL and the Harvard-Oxford cortical and subcortical atlases (Harvard Center for Morphometric Analysis) which are provided with the FSL4 software were used to identify the anatomical characteristics of the resulting PICA maps. The FSL4 fslstats and fslmaths tools were used to calculate the number of non-zero voxels in the selected difference maps, and their *t*-score values.

## Results

**Figure 2:**
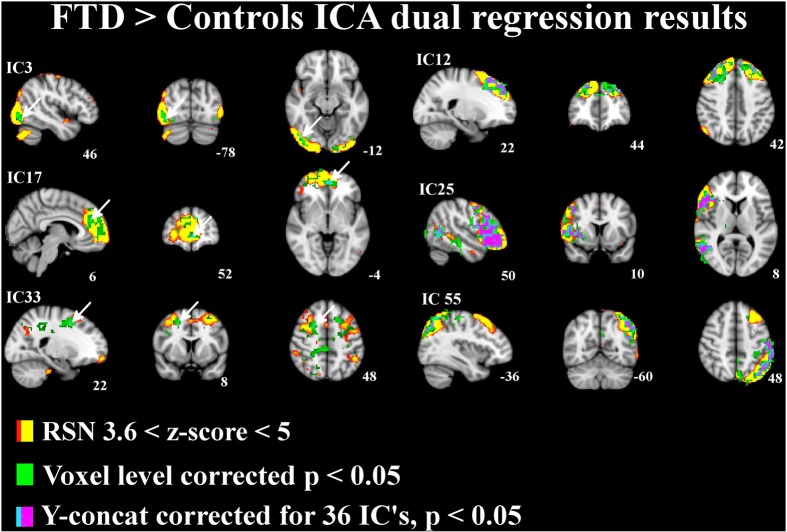
**Independent components (IC) with significant increase in functional connectivity in patients with bvFTD.** The differences in resting state networks corrected for multiple comparison at voxel level in green and for y-concatenation correction for including all RSNs in lilac-blue, threshold *p* < 0.05. The differences between groups are also adjusted for gray matter differences. The involved RSN is presented in red-yellow color encoding with (3.6 < *z*-score < 6) threshold. Arrows in ICs 3, 17, and 33 indicate the y-concatenated results.

The differences between patients with bvFTD and healthy controls were markedly smaller in the dual regression without variance normalization than with normalization. Thus, the differences between groups in the network shape seemed to be relatively limited when gray matter differences were adjusted. The variance normalized and non-normalized dual regression results suggest that the functional connectivity of the baseline RSNs is altered rather than the shape of the network in patients with bvFTD when compared with healthy controls. Based on this we further performed y-concatenation correction for multiple ICA components with the variance normalized (des norm = 1) results. We corrected for both multiple ICs and gray matter volume simultaneously. After this analysis, the number of ICs with significant differences reduced from fifteen detected after only voxel-level correction to six (Table 1, Figure 2).

**Table 1:** **Functional brain regions showing increased functional connectivity in patients with bvFTD compared to healthy controls**.

		**Voxel level correction**								**Inter-IC corrected y-concatenation for all RSNs**								
**IC**	**RSN**	**voxels**	**max**			**min**	**max**	**mean**	**Std**	**IC**	**voxels**	**max**			**min**	**max**	**mean**	**Std**
			***X***	***Y***	***Z***	***T*-score**	***T*-score**	***T*-score**				***X***	***Y***	***Z***	***T*-score**	***T*-score**	***T*-score**	
IC01	Cranial salience, superior frontal gyrus	52	21	42	31	2.63	3.93	3.06	0.33									
IC03	Visual cortex 1–4	103	13	10	14	2.51	4.90	3.36	0.59	IC03	2	11	12	15	4.69	4.81	4.75	0.09
IC04	DAN R	158	9	18	28	2.31	5.26	2.86	0.49									
IC11	Visual cortex, inferior parietal lobule	4	17	9	24	3.59	4.44	3.91	0.35									
IC12	Bilateral frontal pole, middle frontal gyrus	733	29	35	25	1.93	4.86	2.71	0.57	IC12	268	30	38	31	2.60	4.52	3.16	0.38
IC17	Anterior paracingulate	387	22	44	17	2.07	5.05	2.65	0.48	IC17	7	22	45	17	3.76	4.33	4.05	0.21
IC21	Broca L	1369	34	41	14	1.90	5.28	2.91	0.67									
IC25	Insula, inferior frontal gyrus, middle temporal gyrus	1202	11	39	12	1.82	5.53	2.82	0.70	IC25	573	7	18	16	2.49	5.53	3.32	0.61
IC27	Pons, bilateral parahippocampus	7	23	12	21	3.73	4.22	3.97	0.21									
IC33	Premotor-presensory	555	11	32	27	1.91	5.46	2.54	0.49	IC33	2	17	33	30	5.01	5.30	5.16	0.21
IC35	Bilateral primary motor cortex	41	30	24	35	2.45	3.82	2.90	0.35									
IC41	Frontal pole R	508	10	42	13	2.03	6.14	3.20	0.84									
IC47	Primary somatosensory cortex	27	7	29	26	2.86	3.70	3.20	0.21									
IC51	Angular gyrus, bilateral supramarginal gyrus	217	6	22	22	2.40	4.89	3.01	0.47									
IC55	DAN L	956	35	16	24	1.85	5.13	2.53	0.54	IC55	260	24	11	27	2.54	4.58	3.05	0.42

Significant differences in functional connectivity were detected in fifteen RSNs by voxel level correction. By Inter-IC corrected y-concatenation for all RSNs significant differences in functional connectivity were detected in six RSNs. Significant differences are demonstrated by; the anatomical areas involved, number of voxels, MNI coordinates (in mm) of the involved anatomical areas, t-score and its standard deviation. Abbreviations: IC, independent component; R, right; L, left; DAN, dorsal attention network.

Using this protocol, increased connectivity was found in several networks within patients with bvFTD compared to the control group. The most prominent enhancement was seen in the right frontotemporal area and insula (IC 25). Increased connectivity was also detected in the left DAN (IC 55), in the DMN (anterior paracingulate gyrus; IC 17) and in anterior parts of the frontal pole (IC 12). Minor changes were also seen in the visual network in the occipital region (IC 3) and in the premotor-postsensory network (IC 33).

The bvFTD group was divided into two separate groups according to the degree of severity of the disease, mild (*n* = 9) and moderate/severe (*n* = 10). There were no significant differences between mild and moderate to severe subgroups in functional connectivity.

**Figure 3:**
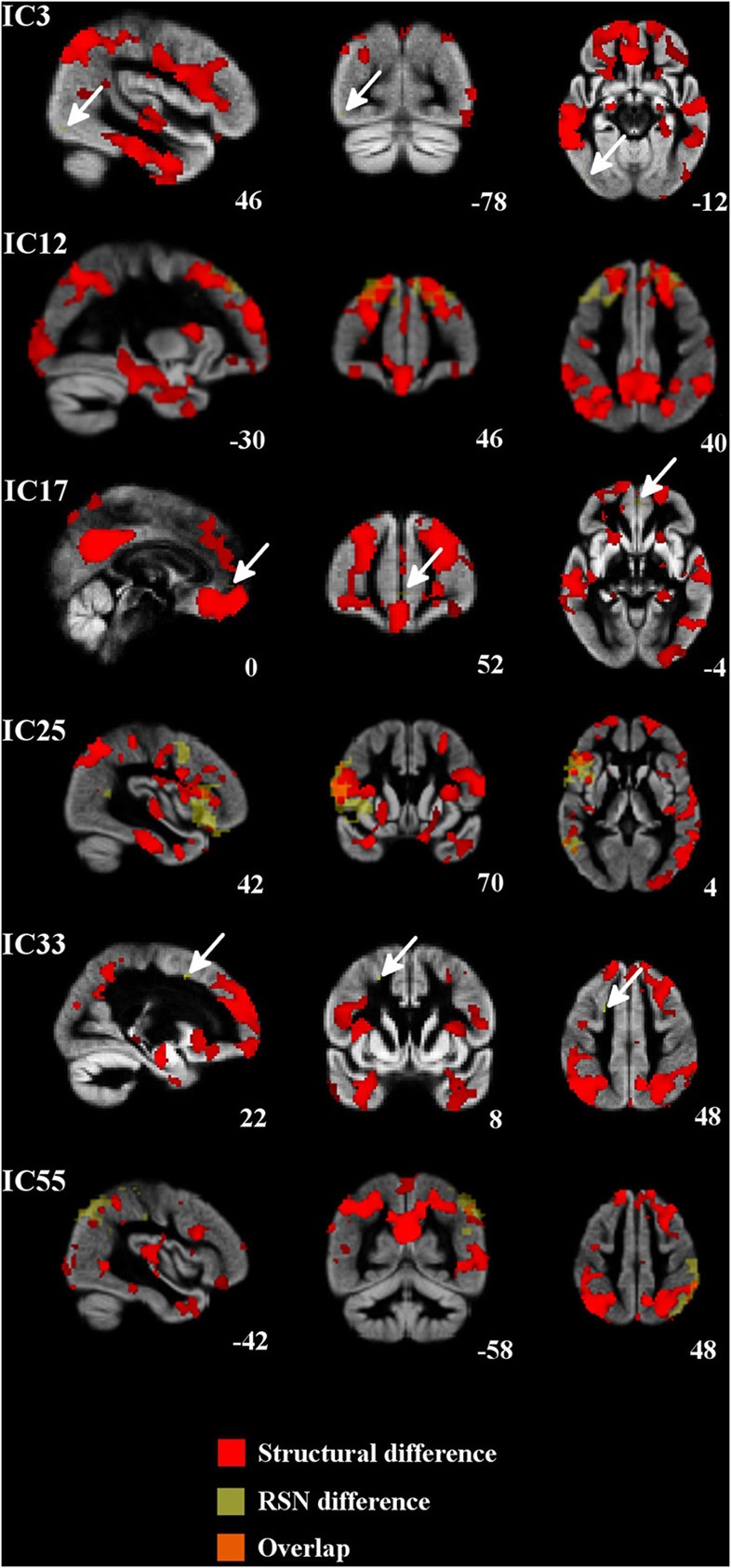
**Regional atrophy was detected in several frontal and temporal regions and also in the posterior cingulate cortex, occipital cortex, and occipital poles.** Increased connectivity was constantly placed around the atrophied area. Atrophic regions are in red, difference in the RSNs are in yellow (bvFTD > controls) and overlapping areas are in orange color. Arrows in ICs 3, 17, and 33 indicate the difference in the RSN.

In structural analysis, widespread gray matter atrophy was detected (Table 2, Figure 3). Atrophy was most prominent in the right temporal lobe in addition to the precuneus cortex and posterior cingulate gyrus. Mild atrophy was also detected in both hippocampi. After the adjustment for gray matter differences, the increased connectivity was predominantly placed around the atrophic area (Figure 3).

**Table 2:** **Regional atrophy detected by structural MRI**.

**Voxels**	**max_*Y***	**min_*Z***	**max_*Z***	**Mean-*T*score**	**sem *T*-score**	**max *T*-score**	**Anatomical area**
16479	72	−52	60	2.94	0.005	5.55	R middle and inferior temporal gyrus
15849	38	−50	72	2.94	0.006	6.38	L precuneus cortex, cingulate gyrus
422	58	−22	−6	2.62	0.016	3.58	L broca, frontal pole, inferior temporal gyrus
278	28	−30	0	3.40	0.045	5.76	L frontal pole, orbitofrontal, and subcallosal cortex
267	18	4	16	3.72	0.050	5.46	L insula, frontal and central opercular cortex, broca
82	−14	60	68	3.68	0.043	4.72	R premotor and motor cortex, precentral gyrus
52	12	−44	−26	2.34	0.020	2.74	R temporal pole, middle temporal gyrus
47	−8	38	46	2.52	0.039	3.24	L postcentral gyrus
42	28	−20	−16	2.39	0.026	2.85	R orbitofrontal cortex, frontal and temporal pole
28	26	−40	−34	2.55	0.062	3.29	R temporal pole
23	−20	48	56	3.43	0.031	3.75	L premotor and primary motor cortex, precentral gyrus
20	−32	−8	−2	3.43	0.048	3.87	R hippocampus, parahippocampal gyrus
19	−32	2	6	2.49	0.054	2.93	L superior and middle temporal gyrus
13	−80	12	14	2.21	0.010	2.28	L lateral occipital cortex

R, right; L, left.

## Discussion

This is the first report of RSNs of the whole brain cortex in patients with bvFTD using a new correction method that allows the assessment of several brain networks. Connectivity changes as well as changes in the shape of the brain networks were analysed. The most extensive increase in connectivity was detected in the frontal poles bilaterally, in the right insular cortex and the ACC in several networks (IC 12, IC 17, IC 25). This area is associated with the human mirror neuron system and the von Economo cells, which are thought to be crucial in social cognition (Seeley et al., 2006, 2007a,b). These cells are destroyed in patients with bvFTD and atrophy in this area is associated with a decline in behavior and loss of empathy and insight in bvFTD. In a recent study, prefrontal hyperconnectivity has been associated with apathy in patients with bvFTD (Farb et al., 2012). Apathy was the most prevalent neuropsychiatric symptom in the present study and may be associated with hyperconnectivity in the prefrontal areas, which confirms the previous findings. Farb and colleagues also detected that prefrontal hyperconnectivity was associated with dementia severity, but this phenomenon was not seen in our patients (Farb et al., 2012).

Impairment in executive functions and attention are prominent neuropsychological findings in bvFTD patients. Significant increase in connectivity was detected in the left DAN (Table 1, Figure 2; IC 55). However, we did not detect any changes in the executive network itself (IC 42), which confirms the previous finding of normal executive networks in patients with bvFTD (Filippi et al., 2012). In previous studies the DAN has been found to be disrupted in AD, but not in bvFTD (Filippi et al., 2012; Li et al., 2012). Despite the normal executive network, a decline in executive functions may be explained by changes in the attention networks and the destruction of several frontal control networks (IC12, 17, 25).

Increased connectivity was also found in the frontal sub-network of the DMN (IC 17). In several studies the disruption of the DMN is consistently associated with AD (Greicius et al., 2004; Zhang et al., 2009; Gili et al., 2011), but the findings are controversial in patients with bvFTD (Zhou et al., 2010; Whitwell et al., 2011; Borroni et al., 2012). In bvFTD, increased DMN connectivity has often been associated with decreased connectivity in the salience network (Zhou et al., 2010; Whitwell et al., 2011; Borroni et al., 2012; Farb et al., 2012). However, in the present study, decreased connectivity was not detected in the salience network even if increased connectivity in the DMN was found.

In structural analyses, the most prominent gray matter atrophy was detected in the right temporal region, precuneus, and posterior cingulate. Atrophy in the posterior cingulate is typically associated with AD and decreased connectivity in DMN. The research reports about gray matter atrophy in posterior brain areas in patients with bvFTD have been controversial. Broadly, precuneus and posterior cingulate seem to be preserved in bvFTD, however gray matter atrophy of these areas has been detected in some studies (Du et al., 2007; Zhou et al., 2010; Hartikainen et al., 2012). In present study increased functional connectivity was consistently located around the atrophic areas. The centrifugal disposition of increased functional connectivity around the atrophic areas suggests plasticity related shifting of function to areas still capable of functioning. It is noteworthy that multi-modal imaging in detecting functional changes in diseases with abnormal anatomy is demanding. For example, how is the variance normalization of the BOLD signal affected by shifted and increased function; is it merely overlap of functional connectivity or plasticity effects? This question currently remains unanswered. In this study we were able to correct our results for both multiple comparisons related to the selecting of 36 RSNs and for gray matter differences known to exist in bvFTD.

We found only increased connectivity in patients with bvFTD compared to controls. Several factors may have an influence upon our results. We have used different data analysing methods compared to the previous rfMRI studies (Zhou et al., 2010; Whitwell et al., 2011; Borroni et al., 2012; Farb et al., 2012; Filippi et al., 2012). Higher model order (a.k.a 70) was used, which yields a more detailed RSN partition and reduces the presence of noise in the maps (Kiviniemi et al., 2009; Smith et al., 2009; Abou-Elseoud et al., 2010). Furthermore, global mean regression may be involved with unpredictable anti-correlations and this may also be one source of differences between our results and others' (Murphy et al., 2009). We also used a group ICA dual regression approach in this study. Matching individual level ICA maps is more prone to errors when compared to group map templates (Zuo et al., 2010). We also share this experience in this dataset. We analysed individual level ICA maps with low (20) model order, but had considerable difficulty in detecting a robustly identifiable salience and other high cognitive networks from individual ICA runs for each subject, especially when compared to group templates. This is not a problem of ICA *per se*, since it has been known to be robust in detecting fMRI sources (Kiviniemi et al., 2009; Smith et al., 2009). Actually, it may be the other way around; ICA is very sensitive to sparsity (Daubechies et al., 2009). On the individual level, the RSNs have marked spatiotemporal non-stationarity due to strong momentary neuronal avalanches (Chang and Glover, 2010; Kiviniemi et al., 2011; Hutchison et al., 2013; Liu and Duyn, 2013; Palva et al., 2013). The neuronal avalanches arising within RSN nodes have unique spatial connectivity distributions and match only with group level RSN templates when strongly averaged in both time and space (Zuo et al., 2010; Liu and Duyn, 2013). Dual regression maps may therefore provide a closer matching representation of an individual subject's RSN compared to ICA based maps.

The advantage of the present method is the whole brain cortex coverage and higher hierarchical level of sub-networks (Kiviniemi et al., 2009; Abou-Elseoud et al., 2010; Littow et al., 2010). Moreover, the high model order enables increased accuracy of regressors of also the unwanted artifacts such as pulsating blood vessels, compartmental CSF pulsation/jet and motion artifacts that cannot be identified with other methods. The regression of these artifacts is crucial for the correct analysis of group differences in any kind of connectivity measurement. ICA dual regression offers a simultaneous data-driven regression of both artifacts and RSNs. Importantly, with group ICA, the effects of curved and often individually different cerebral artery and CSF pulsations become separated by the best way within the analysed group as a whole, also with individual weighting in their time series. The adequate modeling of blood vessel noise may have an effect on the salience network results in the insular cortex near the medial cerebral artery branches. Advanced spiral in/out scanning sequences at the higher 3 T magnetic field are also likely to be more sensitive than conventional 1.5 T EPI sequences in providing frontal cortex/insular data at the individual level (Zhou et al., 2010; Whitwell et al., 2011; Farb et al., 2012; Filippi et al., 2012).

Despite careful diagnostics, long follow-up and genetic confirmation of bvFTD, it is possible that some patients with atypical AD may be included in the study cohort. The wide range in disease severity may affect the rfMRI results even though no difference was seen between the patient groups with different disease severity. Neuroleptic and cholinergic drugs may also modify results. However, there are no rfMRI data available concerning the effects of these drugs.

In conclusion, this is the first analysis of the whole brain cortex networks using high model order ICA in patients with bvFTD. Increased connectivity was seen in the left DAN and frontal control networks neighboring executive network, as well as the frontal poles and insula. These changes may explain bvFTD associated cognitive and neuropsychiatric symptoms. In general, the increase in functional connectivity centrifugally was surrounding the atrophied areas, which suggest plasticity related shifting of neuronal activity into intact brain structures.

### Conflict of interest statement

Dr. Traynor has a patent pending on the diagnostic and therapeutic uses based on the discovery of the hexanucleotide repeat expansion in C9ORF72. The remaining authors declare that the research was conducted in the absence of any commercial or financial relationships that could be construed as a potential conflict of interest.

## References

[B1a] Abou-Elseoud A. Starck T. Remes J. Nikkinen J. Tervonen O. Kiviniemi V. (2010). The effect of model order selection in group PICA. Hum. Brain Mapp. 31, 1207–1216 10.1002/hbm.20929 20063361 PMC6871136

[B1] Abou Elseoud A. Littow H. Remes J. Starck T. Nikkinen J. Nissila J. (2011). Group-ICA model order highlights patterns of functional brain connectivity. Front. Syst. Neurosci. 5:37 10.3389/fnsys.2011.00037 21687724 PMC3109774

[B2] Abou Elseoud A. Nissila J. Liettu A. Remes J. Jokelainen J. Takala T. (2012). Altered resting-state activity in seasonal affective disorder. Hum. Brain Mapp. [Epub ahead of print]. 10.1002/hbm.22164 22987670 PMC6869738

[B3] Abou-Elseoud A. Starck T. Remes J. Nikkinen J. Tervonen O. Kiviniemi V. (2010). The effect of model order selection in group PICA. Hum. Brain Mapp. 31, 1207–1216 10.1002/hbm.20929 20063361 PMC6871136

[B4] Allen E. A. Erhardt E. B. Wei Y. Eichele T. Calhoun V. D. (2012a). Capturing inter-subject variability with group independent component analysis of fMRI data: a simulation study. Neuroimage 59, 4141–4159 10.1016/j.neuroimage.2011.10.010 22019879 PMC3690335

[B5] Allen E. A. Damaraju E. Plis S. M. Erhardt E. B. Eichele T. Calhoun V. D. (2012b). Tracking whole-brain connectivity dynamics in the resting State. Cereb. Cortex. [Epub ahead of print]. 10.1093/cercor/bhs352 23146964 PMC3920766

[B6] Ashburner J. Friston K. J. (2000). Voxel-based morphometry-the methods. Neuroimage 11, 805–821 10.1006/nimg.2000.0582 10860804

[B7] Beckmann C. F. DeLuca M. Devlin J. T. Smith S. M. (2005). Investigations into resting-state connectivity using independent component analysis. Philos. Trans. R. Soc. Lond. B Biol. Sci. 360, 1001–1013 10.1098/rstb.2005.1634 16087444 PMC1854918

[B8] Beckmann C. F. Smith S. M. (2004). Probabilistic independent component analysis for functional magnetic resonance imaging. IEEE Trans. Med. Imaging 23, 137–152 10.1109/TMI.2003.822821 14964560

[B9] Borroni B. Alberici A. Cercignani M. Premi E. Serra L. Cerini C. (2012). Granulin mutation drives brain damage and reorganization from preclinical to symptomatic FTLD. Neurobiol. Aging 33, 2506–2520 10.1016/j.neurobiolaging.2011.10.031 22130207

[B8a] Chang C. Glover G. H. (2010). Time-frequency dynamics of resting-state brain connectivity measured with fMRI. Neuroimage 50, 81–98 10.1016/j.neuroimage.2009.12.011 20006716 PMC2827259

[B10] Daubechies I. Roussos E. Takerkart S. Benharrosh M. Golden C. D'Ardenne K. (2009). Independent component analysis for brain fMRI does not select for independence. Proc. Natl. Acad. Sci. U.S.A. 106, 10415–10422 10.1073/pnas.0903525106 19556548 PMC2705604

[B11] Du A. Schuff N. Kramer J. Rosen H. Gorni-Tempini M. Rankin K. (2007). Different regional patterns of cortical thinning in Alzheimer's disease and frontotemporal dementia. Brain 130, 1159–1166 10.1093/brain/awm016 17353226 PMC1853284

[B12] Farb N. A. Grady C. L. Strother S. Tang-Wai D. F. Masellis M. Black S. (2012). Abnormal network connectivity in frontotemporal dementia: evidence for prefrontal isolation. Cortex 49, 1856–1873 10.1016/j.cortex.2012.09.008 23092697

[B13] Filippi M. Agosta F. Scola E. Canu E. Magnani G. Marcone A. (2012). Functional network connectivity in the behavioral variant of frontotemporal dementia. Cortex. 10.1016/j.cortex.2012.09.017 23164495

[B14] Filippini N. MacIntosh B. J. Hough M. G. Goodwin G. M. Frisoni G. B. Smith S. M. (2009). Distinct patterns of brain activity in young carriers of the APOE-epsilon4 allele. Proc. Natl. Acad. Sci. U.S.A. 106, 7209–7214 10.1073/pnas.0811879106 19357304 PMC2678478

[B15] Fox M. D. Raichle M. E. (2007). Spontaneous fluctuations in brain activity observed with functional magnetic resonance imaging. Nat. Rev. Neurosci. 8, 700–711 10.1038/nrn2201 17704812

[B16] Gili T. Cercignani M. Serra L. Perri R. Giove F. Maraviglia B. (2011). Regional brain atrophy and functional disconnection across Alzheimer's disease evolution. J. Neurol. Neurosurg. Psychiatry. 82, 58–66 10.1136/jnnp.2009.199935 20639384

[B17] Good C. D. Johnsrude I. S. Ashburner J. Henson R. N. Friston K. J. Frackowiak R. S. (2001). A voxel-based morphometric study of ageing in 465 normal adult human brains. Neuroimage 14, 21–36 10.1006/nimg.2001.0786 11525331

[B18] Greicius M. D. Srivastava G. Reiss A. L. Menon V. (2004). Default-mode network activity distinguishes Alzheimer's disease from healthy aging: evidence from functional MRI. Proc. Natl. Acad. Sci. U.S.A. 101, 4637–4642 10.1073/pnas.0308627101 15070770 PMC384799

[B19] Hartikainen P. Rasanen J. Julkunen V. Niskanen E. Hallikainen M. Kivipelto M. (2012). Cortical thickness in frontotemporal dementia, mild cognitive impairment, and Alzheimer's disease. J. Alzheimers Dis. 30, 857–874 10.3233/JAD-2012-112060 22466003

[B20] Hutchison R. M. Womelsdorf T. Allen E. A. Bandettini P. A. Calhoun V. D. Corbetta M. (2013). Dynamic functional connectivity: promises, issues, and interpretations. Neuroimage 15, 360–378 10.1016/j.neuroimage.2013.05.079 23707587 PMC3807588

[B21] Jenkinson M. Bannister P. Brady M. Smith S. (2002). Improved optimization for the robust and accurate linear registration and motion correction of brain images. Neuroimage 17, 825–841 10.1006/nimg.2002.1132 12377157

[B23] Kiviniemi V. Starck T. Remes J. Long X. Nikkinen J. Haapea M. (2009). Functional segmentation of the brain cortex using high model order group PICA. Hum. Brain Mapp. 30, 3865–3886 10.1002/hbm.20813 19507160 PMC6870574

[B24] Kiviniemi V. Vire T. Remes J. Abou Elseoud A. Starck T. Tervonen O. (2011). A sliding time-window ICA reveals spatial variability of the default mode Network in time. Brain Connect. 1, 339–347 10.1089/brain.2011.0036 22432423

[B25] Li R. Wu X. Fleisher A. S. Reiman E. M. Chen K. Yao L. (2012). Attention-related networks in Alzheimer's disease: a resting functional MRI study. Hum. Brain Mapp. 33, 1076–1088 10.1002/hbm.21269 21538702 PMC3150638

[B26] Littow H. Elseoud A. Haapea M. Isohanni M. Moilanen I. Mankinen K. (2010). Age-related differences in functional nodes of the brain cortex - a high model order group ICA study. Front. Syst. Neurosci. 4:32 10.3389/fnsys.2010.00032 20953235 PMC2955419

[B27] Liu X. Duyn J. H. (2013). Time-varying functional network information extracted from brief instances of spontaneous brain activity. Proc. Natl. Acad. Sci. U.S.A. 110, 4392–4397 10.1073/pnas.1216856110 23440216 PMC3600481

[B28] Murphy K. Birn R. M. Handwerker D. A. Jones T. B. Bandettini P. A. (2009). The impact of global signal regression on resting state correlations: are anti-correlated networks introduced? Neuroimage 44, 893–905 10.1016/j.neuroimage.2008.09.036 18976716 PMC2750906

[B29] Neary D. Snowden J. Mann D. (2005). Frontotemporal dementia. Lancet Neurol. 4, 771–780 10.1016/S1474-4422(05)70223-4 16239184

[B30] Neary D. Snowden J. S. Gustafson L. Passant U. Stuss D. Black S. (1998). Frontotemporal lobar degeneration: a consensus on clinical diagnostic criteria. Neurology 51, 1546–1554 10.1212/WNL.51.6.1546 9855500

[B31] Nichols T. E. Holmes A. P. (2002). Nonparametric permutation tests for functional neuroimaging: a primer with examples. Hum. Brain Mapp. 15, 1–25 10.1002/hbm.1058 11747097 PMC6871862

[B32] Palva J. M. Zhigalov A. Hirvonen J. Korhonen O. Linkenkaer-Hansen K. Palva S. (2013). Neuronal long-range temporal correlations and avalanche dynamics are correlated with behavioral scaling laws. Proc. Natl. Acad. Sci. U.S.A. 110, 3585–3590 10.1073/pnas.1216855110 23401536 PMC3587255

[B33] Piguet O. Hornberger M. Mioshi E. Hodges J. R. (2011). Behavioural-variant frontotemporal dementia: diagnosis, clinical staging, and management. Lancet Neurol. 10, 162–172 10.1016/S1474-4422(10)70299-4 21147039

[B34] Raichle M. E. MacLeod A. M. Snyder A. Z. Powers W. J. Gusnard D. A. Shulman G. L. (2001). A default mode of brain function. Proc. Natl. Acad. Sci. U.S.A. 98, 676–682 10.1073/pnas.98.2.676 11209064 PMC14647

[B35] Rascovsky K. Hodges J. R. Knopman D. Mendez M. F. Kramer J. H. Neuhaus J. (2011). Sensitivity of revised diagnostic criteria for the behavioural variant of frontotemporal dementia. Brain 134, 2456–2477 10.1093/brain/awr179 21810890 PMC3170532

[B36] Renton A. E. Majounie E. Waite A. Simon-Sanchez J. Rollinson S. Gibbs J. R. (2011). A hexanucleotide repeat expansion in C9ORF72 is the cause of chromosome 9p21-linked ALS-FTD. Neuron 72, 257–268 10.1016/j.neuron.2011.09.010 21944779 PMC3200438

[B37] Rosen H. J. Gorno-Tempini M. L. Goldman W. P. Perry R. J. Schuff N. Weiner M. (2002). Patterns of brain atrophy in frontotemporal dementia and semantic dementia. Neurology 58, 198–208 10.1212/WNL.58.2.198 11805245

[B38] Rueckert D. Sonoda L. I. Hayes C. Hill D. L. Leach M. O. Hawkes D. J. (1999). Nonrigid registration using free-form deformations: application to breast MR images. IEEE Trans. Med. Imaging 18, 712–721 10.1109/42.796284 10534053

[B39] Seeley W. W. Carlin D. A. Allman J. M. Macedo M. N. Bush C. Miller B. L. (2006). Early frontotemporal dementia targets neurons unique to apes and humans. Ann. Neurol. 60, 660–667 10.1002/ana.21055 17187353

[B40] Seeley W. W. Allman J. M. Carlin D. A. Crawford R. K. Macedo M. N. Greicius M. D. (2007a). Divergent social functioning in behavioral variant frontotemporal dementia and Alzheimer disease: reciprocal networks and neuronal evolution. Alzheimer Dis. Assoc. Disord. 21, S50–S57 10.1097/WAD.0b013e31815c0f14 18090425

[B41] Seeley W. W. Menon V. Schatzberg A. F. Keller J. Glover G. H. Kenna H. (2007b). Dissociable intrinsic connectivity networks for salience processing and executive control. J. Neurosci. 27, 2349–2356 10.1523/JNEUROSCI.5587-06.2007 17329432 PMC2680293

[B42] Smith S. M. (2002). Fast robust automated brain extraction. Hum. Brain Mapp. 17, 143–155 10.1002/hbm.10062 12391568 PMC6871816

[B43] Smith S. M. Fox P. T. Miller K. L. Glahn D. C. Fox P. M. Mackay C. E. (2009). Correspondence of the brain's functional architecture during activation and rest. Proc. Natl. Acad. Sci. U.S.A. 106, 13040–13045 10.1073/pnas.0905267106 19620724 PMC2722273

[B44] Veer I. M. Beckmann C. F. van Tol M. J. Ferrarini L. Milles J. Veltman D. J. (2010). Whole brain resting-state analysis reveals decreased functional connectivity in major depression. Front. Syst. Neurosci. 4:41 10.3389/fnsys.2010.00041 20941370 PMC2950744

[B45] Whitwell J. L. Josephs K. A. Avula R. Tosakulwong N. Weigand S. D. Senjem M. L. (2011). Altered functional connectivity in asymptomatic MAPT subjects: a comparison to bvFTD. Neurology 77, 866–874 10.1212/WNL.0b013e31822c61f2 21849646 PMC3162637

[B46] Whitwell J. L. Przybelski S. A. Weigand S. D. Ivnik R. J. Vemuri P. Gunter J. L. (2009). Distinct anatomical subtypes of the behavioural variant of frontotemporal dementia: a cluster analysis study. Brain 132, 2932–2946 10.1093/brain/awp232 19762452 PMC2768663

[B47] Zhang H. Y. Wang S. J. Xing J. Liu B. Ma Z. L. Yang M. (2009). Detection of PCC functional connectivity characteristics in resting-state fMRI in mild Alzheimer's disease. Behav. Brain Res. 197, 103–108 10.1016/j.bbr.2008.08.012 18786570

[B48] Zhang Y. Brady M. Smith S. (2001). Segmentation of brain MR images through a hidden Markov random field model and the expectation-maximization algorithm. IEEE Trans. Med. Imaging 20, 45–57 10.1109/42.906424 11293691

[B49] Zhou J. Greicius M. D. Gennatas E. D. Growdon M. E. Jang J. Y. Rabinovici G. D. (2010). Divergent network connectivity changes in behavioural variant frontotemporal dementia and Alzheimer's disease. Brain 133, 1352–1367 10.1093/brain/awq075 20410145 PMC2912696

[B50] Zuo X. N. Kelly C. Adelstein J. S. Klein D. F. Castellanos F. X. Milham M. P. (2010). Reliable intrinsic connectivity networks: test-retest evaluation using ICA and dual regression approach. Neuroimage 49, 2163–2177 10.1016/j.neuroimage.2009.10.080 19896537 PMC2877508

